# Characterization of cast Ti_30_Cr_20_Mo_15_Zr_10_Ta_5_Nb_20-x_Fe_x_ compositionally complex alloys

**DOI:** 10.1038/s41598-026-54590-1

**Published:** 2026-05-26

**Authors:** Aya A. Ibrahim, Lamiaa Z. Mohamed, Mohamed El-shazly, M. El Sherbiny, Shimaa El-Hadad

**Affiliations:** 1 Faculty of Engineering, Cairo University, Giza, 12613 Egypt; 2 Mechatronics Engineering Department, Canadian International College, Cairo, Egypt; 3 Central Metallurgical Research and Development Institute (CMRDI), P.O. 87, Helwan, Egypt

**Keywords:** Compositionally complex alloys, Microstructure, Wear resistance, Corrosion, Engineering, Materials science

## Abstract

This study systematically investigates the relationship between the microstructure and performance of two cast Ti_30_Cr_20_Mo_15_Zr_10_Ta_5_Nb_20-x_Fe_x_ compositionally complex alloys (CCAs), prepared by vacuum arc melting. In the first alloy with (x = 0.0), 20 at.% Nb was added, resulting in the composition of Ti_30_Cr_20_Mo_15_Zr_10_Ta_5_Nb_20_ CCA (20Nb), while in the second version (x = 10), a more cost-effective variant was developed by partially substituting Nb with 10 at.% Fe, yielding the composition of Ti_30_Cr_20_Mo_15_Zr_10_Ta_5_Nb_10_Fe_10_ CCA (10Fe10Nb). Microstructural analysis showed that both alloys have a dendritic structure, with BCC1 as the main phase and a minor BCC2 phase. Some intermetallic phases, such as ZrCr_2_, MoNb, and MoTa, were also observed in the 20Nb alloy. In the Fe-containing CCA, more intermetallic compounds were formed with Zr, Cr, Ta, and Ti. The partial replacement of Nb with Fe in the 10Fe10Nb alloy reduced the intensity of the solid solution phases and promoted the formation of additional intermetallic compounds. The microstructure in both alloys was dendritic, with segregation of high-melting-point elements to the dendritic regions. In terms of mechanical properties, the 20Nb alloy exhibited a lower hardness (584 HV) than 10Fe10Nb (667 HV). The 10Fe10Nb alloy demonstrated a higher Young’s modulus of (102.47 GPa), while the 20Nb alloy measured (85.92 GPa). Regarding corrosion resistance in saline solution, the 20Nb alloy provided better corrosion protection than 10F10Nb without hydroxyapatite (HA) addition. However, both alloys showed excellent corrosion resistance in the presence of 3 g of HA inhibitor. The corrosion rate of 20Nb decreased from 39.09 μm/y without HA to 1.94 μm/y with 3 g HA, and that of 10Fe10Nb reduced from 61.84 μm/y without HA to 0.38 μm/y with 3 g HA. This emphasizes the effective interaction between Fe–Nb oxides and the deposited HA. Moreover, the incorporation of Nb promoted the formation and stabilization of a passive layer composed of Nb_2_O_5_ and NbO_2_, while the addition of HA further enhanced the film’s thickness and compactness. Concluding, the properties of Ti_30_Cr_20_Mo_15_Zr_10_Ta_5_Nb_20-x_Fe_x_ CCAs can be tailored for a specific application through balancing Nb and Fe contents, and lower-cost versions can be produced.

## Introduction

The field of biomaterials currently focuses on developing metallic implants, characterized by an optimal balance between strength and plasticity, for use in orthopedic and cardiovascular stents^1,2^. While titanium, stainless steel, and cobalt-chromium-based alloys dominate clinical use, they face significant limitations^3^. Titanium alloys, for instance, suffer from inadequate wear resistance, which leads to debris release and “particle disease” in physiological environments^4,5^. The critical mode of failure for these implants is corrosive wear, a synergistic process in which corrosion and mechanical wear in body fluids accelerate material degradation and shorten service life. Consequently, developing new alloys that simultaneously offer excellent biocompatibility, high corrosion resistance, and superior wear resistance is a key scientific challenge^6,7^.

High-entropy alloys (HEAs) have emerged as compelling candidates to meet this challenge. Defined by their multi-principal-element composition (typically five or more elements), HEAs exhibit unique “core effects,” such as high configurational entropy and severe lattice distortion^8,9^. These effects are known to produce exceptional mechanical and anti-corrosion properties that surpass those of traditional alloys, positioning HEAs as highly promising candidates for next-generation biomedical implants^10^. Over recent decades, diverse high-entropy alloy (HEA) systems have been successfully developed, including those based on transition, refractory, and eutectic elements, as well as high-entropy metallic glasses^11,12^. Among these, Ti-Zr-Nb-based refractory HEAs have acquired significant interest in biomedical applications due to the inherent biocompatibility of their constituent elements. This led to the development of several biocompatible systems, such as Ti-Zr-Nb-Ta, Ti-Zr-Nb-Mo-Ta, and Ti-Ni-Cr-Fe-Co alloys. These Ti-Zr-Nb-based HEAs typically possess a body-centered cubic (BCC) solid-solution structure^13–15^. This microstructure confers superior hardness, yield strength, and wear resistance compared with conventional alloys such as Ti6Al4V. Their high strength provides excellent resistance to plastic deformation and fracture under load, while their hardness reduces the risk of wear debris generation and associated “particle disease” in physiological environments^7^. Furthermore, the refractory elements (Ti, Zr, Nb, Ta) readily form a dense, protective surface oxide layer, thereby enhancing corrosion resistance^15,16^.

Ti-Zr-Nb-based HEAs exhibit a more favorable combination of properties than traditional metallic biomaterials, making them potential alternatives for implant applications. To date, research has produced various systems within this family, though most are equimolar. However, microstructure and properties are highly sensitive to compositional variations. For example, the non-equimolar Ti_0.5_ZrNbTaMo HEA demonstrates higher yield strength and wear resistance than its equimolar TiZrNbTaMo counterpart^15^.

Following the seminal work by Senkov et al.^17^ on single-phase BCC refractory HEAs (WNbMoTa and WNbMoTaV), subsequent research has expanded to include other BCC systems incorporating early transition metals like zirconium^18^. The ions released from elements such as zirconium, niobium, and tantalum are chemically similar to titanium ions and are generally non-toxic in biological environments. This biocompatibility arises because these active ions readily form oxides, hydroxides, or inorganic salts by bonding with surrounding water molecules or anions^19^.

In recent years, the promising biomaterial concept of the MoNbTaTiZr system has spurred studies on both its equiatomic and non-equiatomic compositions^19,20^. This alloy family has been explicitly designed of elements with low biotoxicity and is currently under investigation for surgical instruments and orthopedic implants^21^. To further explore this compositional space, a variant alloy, FeMoTaTiZr, was developed by substituting niobium for iron^22^. This substitution was motivated by two factors: iron is essential to human metabolism and does not tend to accumulate in tissues^23^, and its lower melting point (1538 °C vs. 2477 °C for Nb) promotes better dissolution and homogeneity during arc melting.

A comparative study by Miguel et al.^24^ investigated the microstructure and corrosion resistance of the two non-equiatomic HEAs (MoNbTaTiZr and FeMoTaTiZr) in a simulated physiological environment. The analysis revealed that, while both alloys exhibited similar corrosion mechanisms and product layers, as suggested by their identical equivalent electrical circuit model, the FeMoTaTiZr alloy (Fe-HEA) exhibited a higher corrosion rate than the Nb-containing variant (Nb-HEA). The authors attributed this to the lower stability of iron species, which likely facilitates selective dissolution and accelerates corrosion. Wang et al.^25^ investigated TiZrHfNbFe HEAs, which exhibited a dendritic microstructure consisting of BCC and Laves dual phases. The dendrites were primarily BCC, while the interdendritic regions were rich in the Laves phase. They found that increasing the Fe content refined the dendrites and increased the volume fraction of the Laves phase. This microstructural evolution led to a significant increase in microhardness, from 310 HV in the Fe0 alloy to 770 HV in the Fe2 alloy. Among the studied compositions, the Fe0.5 alloy demonstrated optimal comprehensive properties, including a high yield strength of 1450 MPa and approximately 8% plastic strain. The corrosion performance of this alloy was also notable, with a high corrosion potential of −0.30 V and a low corrosion current density of 2.80 × 10^−7^ A·cm^−2^. Furthermore, no corrosion pits were observed on the Fe0.5 alloy after polarization, indicating superior stability of the passive film.

Based on the literature on HEAs, the addition of Fe to Ti-based HEAs enhances mechanical properties, improves corrosion/wear resistance, and reduces production costs. However, the HEA systems containing elements such as Cr, Mo, Zr, Ta, Nb, and Fe remain underexplored. Existing studies have focused on phase formation and basic mechanical properties, with insufficient attention given to the integrated effects of compositional variation on both mechanical performance and electrochemical behavior in physiologically relevant environments. Moreover, the role of HA, a key bioceramic, in influencing the corrosion resistance of these alloys remains poorly understood.

To address these gaps, the present study synthesizes and characterizes two novel Ti-based compositionally complex alloys (CCAs): Ti_30_Cr_20_Mo_15_Zr_10_Ta_5_Nb_20_, and its economical version containing Fe: Ti_30_Cr_20_Mo_15_Zr_10_Ta_5_Nb_10_Fe_10_ CCAs. CCAs are characterized by microstructural flexibility, enabling the coexistence of intermetallic phases and solid solutions^26,27^. The design approach of CCAs was recently proposed to address the limitations and complexity of HEAs^27,28^. The CCAs in the current work are based on tailored Nb/Fe incorporation and are supported by a comprehensive evaluation combining mechanical properties and electrochemical analyses.

## Experimental work

### Alloys design

**Fig. 1:**
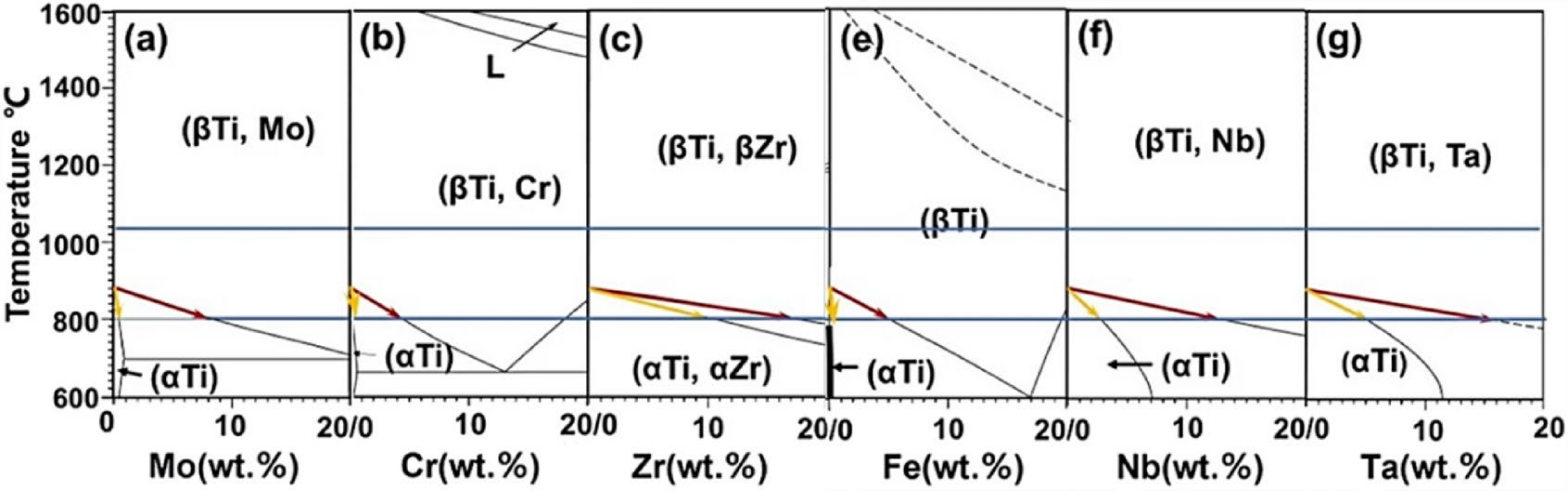
Binary phase diagrams of the β-stabilizing elements^31^.

In designing a Ti-based alloy, generally, the first thing to consider is the influence of the additives on the transformation temperature from alpha (α) to beta (β) phase^29^. The alpha phase has a hexagonal close-packed (HCP) structure, while the beta phase is the body-centered cubic (BCC) allotropic form of titanium^30^. To stabilize the β phase and achieve higher ductility and controllable strength, the type and number of stabilizers should be considered. Figure 1 shows the binary phase diagrams of the β-stabilizing elements included in the current investigation, adopted from^31^.

**Fig. 2:**
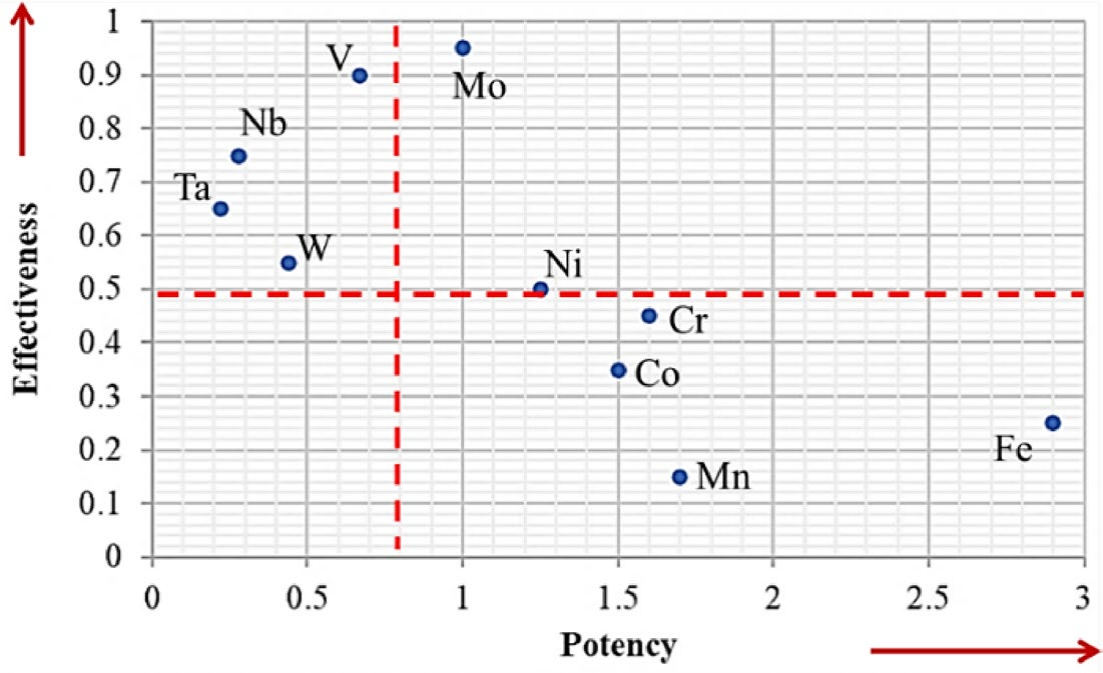
Ranking beta phase stabilizers in terms of potency and effectiveness.

Some of these elements are more effective in β-stabilization, while others are more potent. Effectiveness refers to the overall engineering performance of the final alloy in its intended use. At the same time, potency is the stabilizing power, i.e., the amount of beta stabilizer required to retain the beta phase upon quenching^29–31^. Based on the literature^32,33^, Fig. 2 illustrates the elements used in the current design. Isomorphous stabilizers, such as Mo, Nb, and Ta, have unlimited solubility in beta-Ti and slow eutectoid decomposition. On the other hand, Eutectoid formers, such as Fe and Cr, have limited solubility and form intermetallic compounds, making them potent but potentially detrimental to ductility if not controlled. In the current work, we aimed at choosing the reported set of elements (Ti, Cr, Mo, Zr, and Ta) to prepare two CCAs. In the first alloy, Nb, an effective beta-stabilizing element, was used (20Nb CCA). A second cost-effective alloy containing Fe and Nb was prepared (10Fe10Nb CCA).

To promote solid solution formation, the compositions of 20Nb-, and 10Fe10Nb-CCAs were selected based on Hume-Rothery rules. Their phase formation likelihood was assessed through theoretical parameters (Table 1), such as the enthalpy of mixing (∆H_mix_), the atomic size difference (δ), the solid-solution formation parameter (Ω), and the valence electron concentration (VEC). The results confirm that all alloys meet the common criteria for solid solution formation (i.e., − 15 < ∆H_mix_ < 5 kJ/mol^8^). Notably, their low VEC values (< 6.87) consistently favor a BCC phase^9^. Further analysis of the thermodynamic data reveals distinct profiles:

- 20Nb-CCA: shows the highest thermodynamic drive for a stable solid solution (high Ω, high melting temperature (Tm), and low δ), which is expected to enhance their stability at high-temperature applications^25^.
- 10Fe10Nb-CCA: displays properties, balancing the characteristics of its constituent elements for potential use in multifunctional applications.

**Table 1:** The thermodynamics data of the cast 20Nb and 10Fe10Nb-CCAs.

Code	∆H_mix_ (kJ/mol)	∆S_mix_ (J/K mol)	VEC	δ, %	T_m_ (K)	Ω	∆S_mix_ /R
20Nb	− 6.07	13.88	4.95	7.14	2478.95	5.67	1.67
10Fe10Nb	− 9.13	15.03	5.25	8.07	2335.05	3.84	1.81

Referring to Table 1, ΔH_mix_ is − 6 kJ/mol and − 9 kJ/mol for 20Nb and 10Fe10Nb, respectively. According to the literature^34^, alloys with lower ΔHmix values are more susceptible to forming intermetallic compounds than their counterparts. This means that 10Fe10Nb CCA is expected to contain more intermetallic phases than the 20Nb alloy. Taking into consideration that Table 1 shows a theoretical design, based on the assumption that the CCAs analyzed are representative of the intended design. Consequently, some variation in the microstructure of the actual cast samples is to be expected.

### Materials manufacturing

**Fig. 3:**
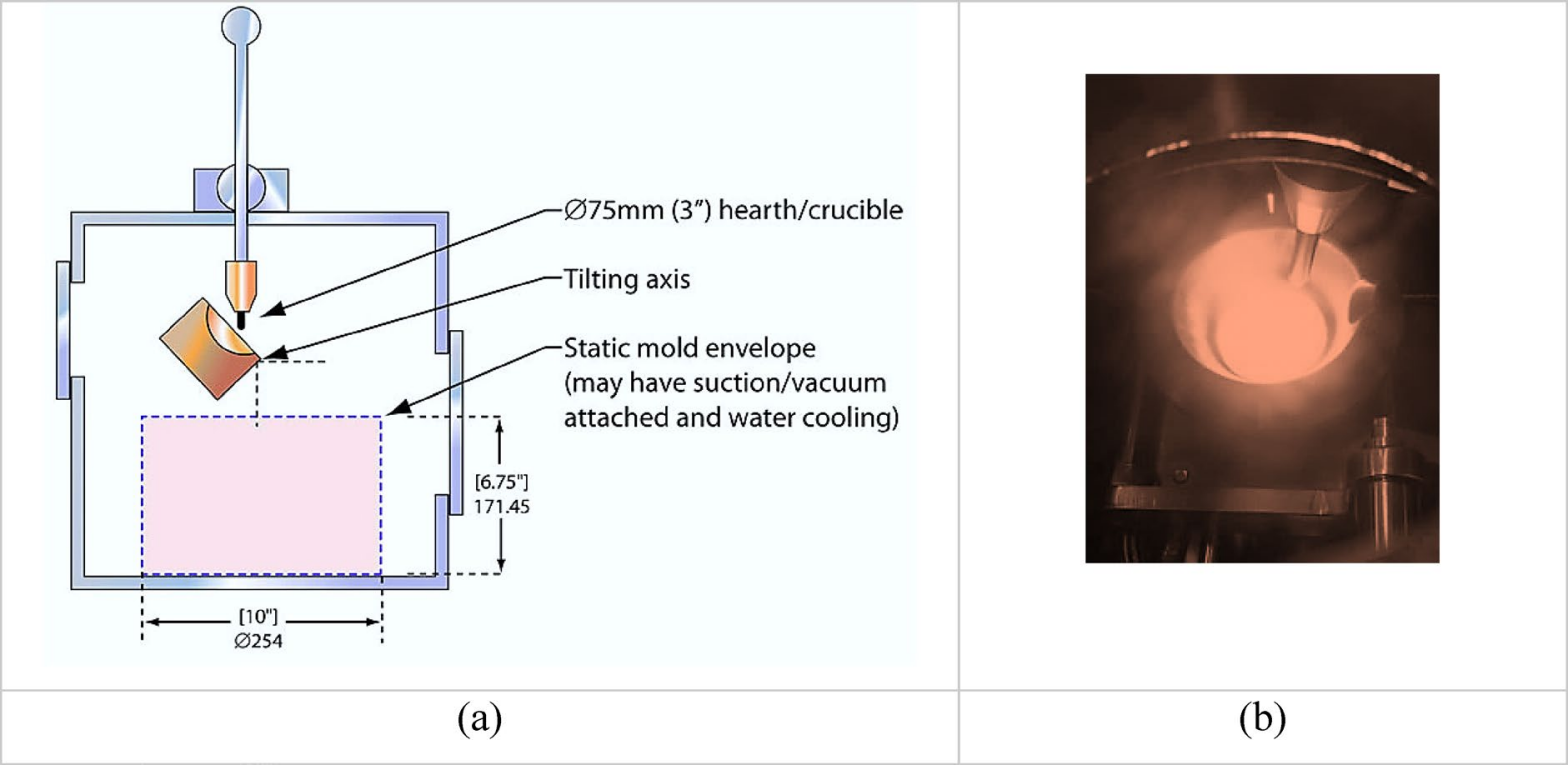
(**a**) A schematic presentation and (**b**) the actual picture of the arc melting process.

High-purity metals were synthesized to prepare (20Nb) and (10Fe10Nb) CCAs. An arc-melting furnace (ARCAST Inc., ME, USA), equipped with a vacuum pump that reaches a maximum pressure of (1.6 × 10^−3^ mbar), was used. The melting chamber is made of stainless steel with a water-cooled hemispherical copper crucible. A tungsten electrode was used to generate the arc. In this melting process, the charge (100 g weight) is placed in the crucible before evacuation. After the vacuum level is reached, argon is introduced into the vacuum chamber, and a high voltage is then applied to create the arc between the charge and the electrode. In the current work, magnetic stirring was applied during melting, and each alloy was remelted five times to guarantee homogeneous composition. A schematic of the vacuum arc melting process is shown in Fig. 3.

### Materials characterization

#### Phase identification and microstructure

Ti-based CCA samples were sectioned using a wire-cut electrical discharge machine to maintain dimensional accuracy and minimize thermal oxidation. The specimens were then sequentially ground with silicon carbide (SiC) abrasive papers up to a 1200-grit finish, followed by final polishing with a 0.3 µm alumina suspension to achieve a mirror-like surface. For microstructural examination, the sample dimensions were 1 cm × 1 cm × 0.5cm, with the polished surfaces etched with a solution of 8% HF, 7% HNO₃, and 85% distilled water (by volume). Microstructural observation was performed using optical microscopy (OM; Olympus DSX1000, operated with PRECiV DSX software). Scanning electron microscopy (SEM) and elemental analysis were conducted using a field-emission SEM (Quanta FEG 250, FEI) equipped with energy-dispersive X-ray spectroscopy (EDX). Phase identification was carried out via X-ray diffraction (XRD) on a PANalytical X’Pert PRO diffractometer with Cu Kα radiation (λ = 0.15406 nm) using a sample with dimensions 1 cm × 1 cm × 0.3 cm.

#### Mechanical Properties of the Investigated CCAs

Vickers microhardness was measured using a Heinrich tester with a 5 N load and a 10 s dwell time, and the reported value represents the average of five indentations on a 1 cm × 1 cm × 0.5 cm sample. Fracture toughness was estimated from the hardness indentations using the indentation fracture method, reported in the work of Nihara et al.^35^.

Wear resistance was evaluated using a pin-on-disc configuration on a T-01 M tribometer under a normal load of 6 kg, a sliding speed of 0.5 m/s, and a total distance of 900 m with a sample dimension of 8 mm in diameter and 1 cm in height. The counterpart was a steel disc with a hardness of 65 HRC. Both the coefficient of friction (COF) and the sample weight loss were recorded in real time during the tests. The COF was determined using Eq. (1), as described in references^12,16,28^.

1 $${\mathrm{COF}} = {\mathrm{Ft}}/{\mathrm{Fn}}$$

In this equation, F_t_ represents the tangential friction force, while F_n_ denotes the applied normal load in Newtons^12^. The specific wear rate (SWR, mm^3^/N·m) was measured for all tested samples.

#### Young’s modulus

**Fig. 4:**
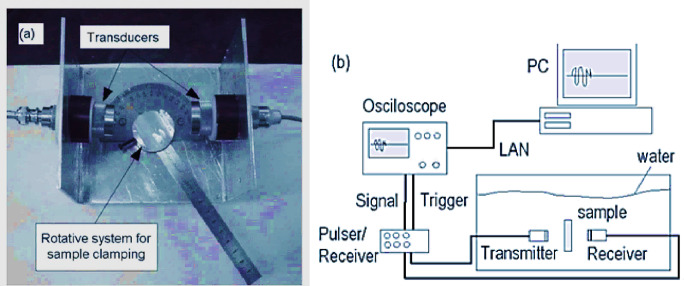
Physical measurements of Young’s Modulus (**a**) Photograph of the mechanical system, (**b**) Block diagram^36^.

The Young’s modulus of the 20Nb and 10Fe10Nb- CCAs was measured at room temperature using the pulse-echo ultrasonic technique with a 5 MHz transducer. Measurements were performed using a Mettler H72 analytical balance with a maximum capacity of 160 g and an accuracy of 0.1 mg. An ultrasonic defect detector (Model: USM3) was used to analyze rectangular specimens (1 cm × 0.5 cm × 0.5 cm) (Fig. 4). This method has been reported to provide versatile, precise measurements of the elastic properties of isotropic materials.

The density (ρ) required for calculations was determined gravimetrically. The ultrasonic defect detector determined the sound velocity (v). Based on ρ and v, the longitudinal modulus (L), shear modulus (G), bulk modulus (K), and Poisson’s ratio (σ) were calculated using the equations reported in^36,37^. Finally, Young’s modulus was calculated according to Eq. (2)^37^:

2 $${\mathrm{E}} = {2}\left( {{1} + \sigma } \right){\mathrm{G}}$$

#### Corrosion test

Corrosion tests were conducted with different concentrations of hydroxyapatite (HA) (0, 1, 2, and 3 g) in saline solutions (0.9 wt.% NaCl, pH about 5.5). HA was supplied as a suspended particulate phase, since it is poorly soluble in aqueous solution at neutral pH. To ensure uniform dispersion throughout the experiment, HA powder was continuously stirred with a magnetic stirrer during dissolution in saline. Both cathodic and anodic scans were conducted at room temperature (RT) to detect potentiodynamic polarization (PDP). The CCA sample was used as the working electrode, the reference electrode was a saturated calomel electrode (SCE), and the counter electrode was a platinum sheet. An Autolab PGSTAT 302N potentiostat with a potential step of 0.001 V and a scan rate of 2 mV/s was used to perform the electrochemical tests for 5400 S. The test specimen dimensions were 1 cm by 1 cm by 0.5 cm. For saline solutions with and without HA, key electrochemical parameters, including corrosion potential (Ecorr), corrosion current density (Icorr), and corrosion rate (CR), were measured. Equation (3) was used to get the corrosion rate^28^:

3 $${\mathrm{CR}}\left( {{\mathrm{mm}}/{\mathrm{y}}} \right) = {3}.{27} \times {1}0^{{ - {3}}} \times {\mathrm{Icorr}} \times {\mathrm{EW}}/\rho$$

where ρ is the HEA’s density (in g/cm^3^), Icorr (in µA/cm^2^) represents the corrosion current density, and EW is the equivalent weight of the HEAs.

## Results and discussion

### Phases identification

In the current work, theoretical Al-equivalent (Aleq) and Mo-equivalent (Moeq) values were calculated to predict the phase stability of the designed alloys. Although current CCA aren’t like traditional titanium alloys, the same concept applies^38^. Using the Aleq–Moeq concept, the crystal structure (BCC or HCP) could be estimated in several commercial Ti-based alloys^29^. Table 2 presents the chemical composition of the alloys and the corresponding Aleq and Moeq values. Following the (Aleq. Vs. Moeq.) diagram by Attallah et al.^39^, a single BCC phase is expected.

**Table 2:** Chemical composition of the prepared CCAs and their corresponding Aleq and Moeq.

Code	Elements wt.%										
	Ti	Cr	Mo	Zr	Ta	Nb	Fe	[Al] eq	[Mo] eq	Avg. Bo	Avg. Md
20Nb	18.9	13.7	19	12	11.9	24.5	0	2	50.398	2.93785	2.2286
10Fe10Nb	19.9	14.4	19.9	12.6	12.5	12.9	7.7	2.1	71.632	2.89305	2.0831

To further confirm the existing phases, the molecular orbital diagram method was used^40^. In this method, two parameters are identified and calculated: Bo, the bond order representing the strength of the covalent bond between an alloying element and titanium, and Mo, the energy level of the d-metal transition elements. The following equations were adopted:^41^

4 $$\overline{Bo }={\sum}_{i}^{n}{x}_{i}{\left(Bo\right)}_{i}$$

5 $$\overline{Md }={\sum}_{i}^{n}{x}_{i}{\left(Md\right)}_{i}$$

**Fig. 5:**
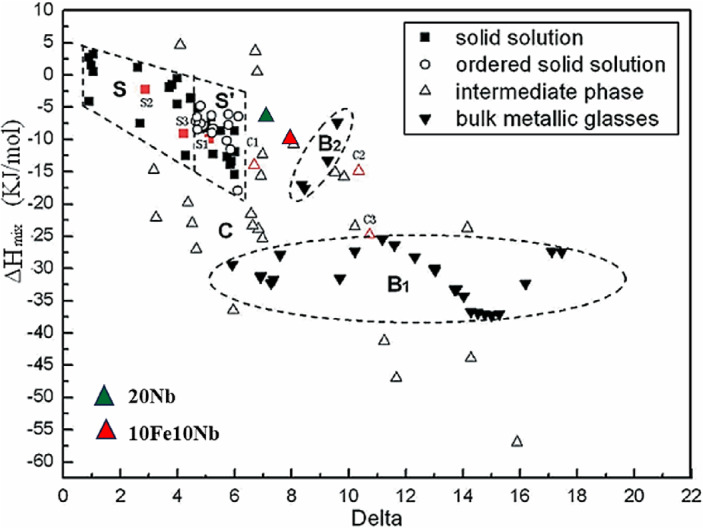
The prepared CCAs are shown as colored triangles [reproduced from Ref. 34, with permission].

Following the Bo-Md diagram of Abdel-Hady et al.^42^, the calculated average values of Bo-Md in Table 2 were located, and the expected phases were determined. According to this diagram, both alloys are fully within the β (BCC) zone, where relatively low E and lower brittleness are expected. This further confirms that the BCC solid solution is the main phase in the two CCAs. Another approach reported by Zhang et al.^34^ was considered to investigate the SS and brittle (glassy) phases in HEAs. A curve of ∆H_mix_ is plotted against “δ” to show the relationship between multicomponent HEAs and multicomponent bulk metallic glasses (MBMGs). Five zones were identified, zone S: which is the solid solution zone where the difference in the atomic size of the constituting elements is small and no enough ∆H_mix_ to form compounds; zone S’: is also a SS but sometimes precipitation of ordered SS occurs due to increased value of δ; Zone B1 is for MBMGs such as Zr-based ones; Zone B2 is for MBMGs based on Mg and Cu and finally Zone C: that represent several phases for HEAs. In contrast to HEAs, MBMGs have more negative ∆H_mix_ and larger δ. Figure 5 locates the current CCAs in their respective zones on the diagram by Zhang et al.^34^. Remarkably, the prepared CCAs are entirely positioned within the intermediate phase region. This indicates that intermetallic compounds may coexist with the BCC solid-solution phase, particularly in the Fe-containing alloy (10Fe10Nb-CCA).

**Fig. 6:**
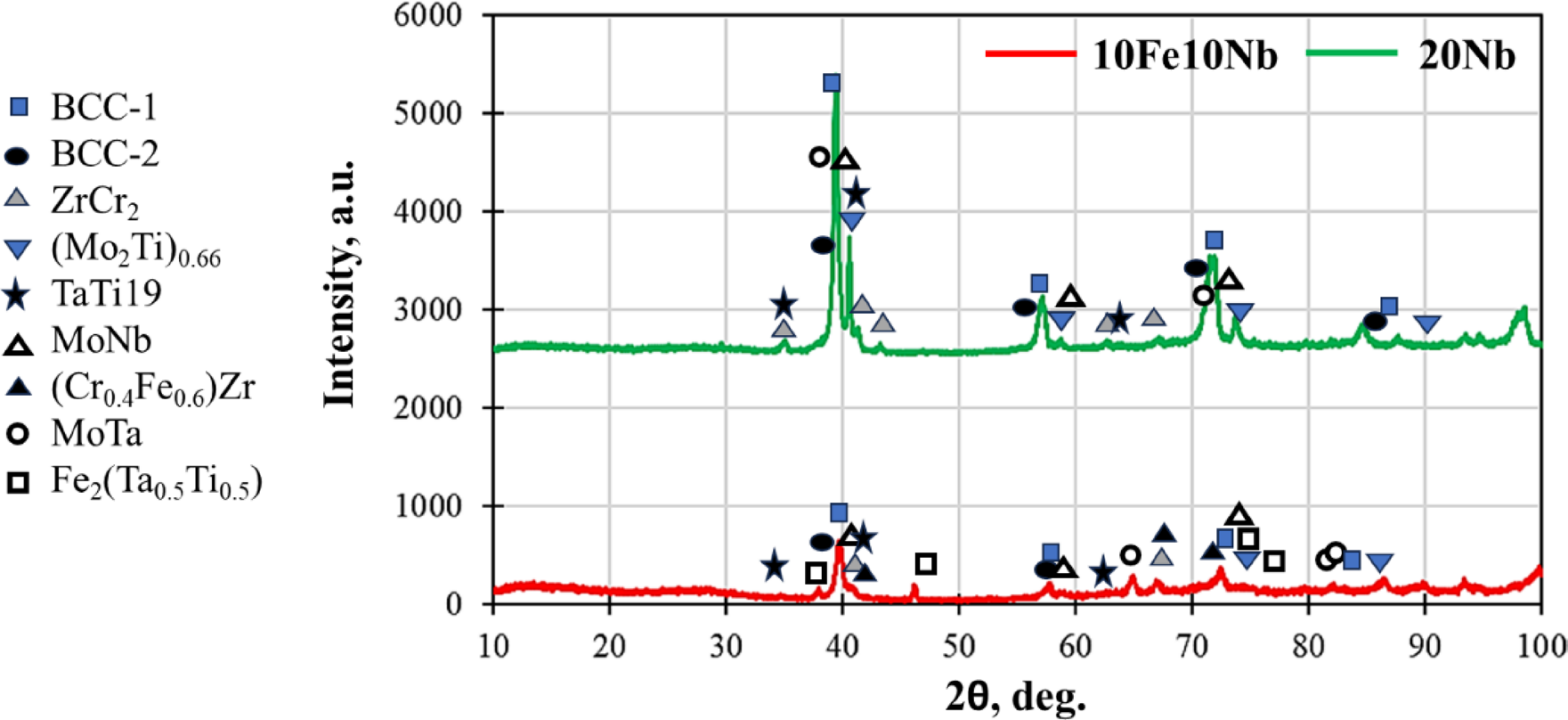
The XRD of cast 20Nb- and 10Fe10Nb-CCAs.

Figure 6 illustrates the XRD patterns of the cast CCAs, highlighting the pronounced compositional influence of Nb and Fe on the constituting phases. The results indicate the coexistence of a predominant BCC1 phase (major phase) at (2θ = 39.383, 56.917, and 71.411 degrees) and a minor BCC2 phase, as depicted in Fig. 6 and Table 3. The diffraction peaks of the BCC2 phase appeared as shoulders on the peaks of the BCC1 phase at slightly lower Bragg angles (at 2θ = 39.36, 56.888 , and 71.373 degrees), suggesting that the lattice parameter of the BCC2 phase is marginally larger than that of the BCC1 phase. Wang and Xu^43^ in their work on TiZrNbTaMo HEAs, deconvoluted the most intense diffraction peaks using Lorentz functions and analyzed their integrated intensities, estimating the volume fraction of the minor BCC2 phase to be approximately 30% and that the lattice parameters of BCC and BCC2 are 0.3310 nm and 0.3379 nm, respectively. In contrast to this reported work, the current CCAs have elemental Cr and Fe. In Fig. 6, several intermetallics coexist with the BCC solid solutions. This agrees with the analysis of the results based on the reported literature^34,39,42^. The presence of these intermetallic phases distinguishes the CCAs from HEAs, in which the greater flexibility of alloying additives enables the attainment of specific properties^44^. It’s obvious that Fe was more interactive with the other elements, thus forming several intermetallic compounds in the 10Fe10Nb alloy, and the peaks for SS hence showed less intensity than in the 20Nb CCA.

**Table 3:** The main observed peaks for SS phases in the prepared CCAs.

hkl	110	200	211	
BCC1-SS	39.383	56.917	71.411	2θ
BCC2-SS	39.36	56.888	71.373	

**Fig. 7:**
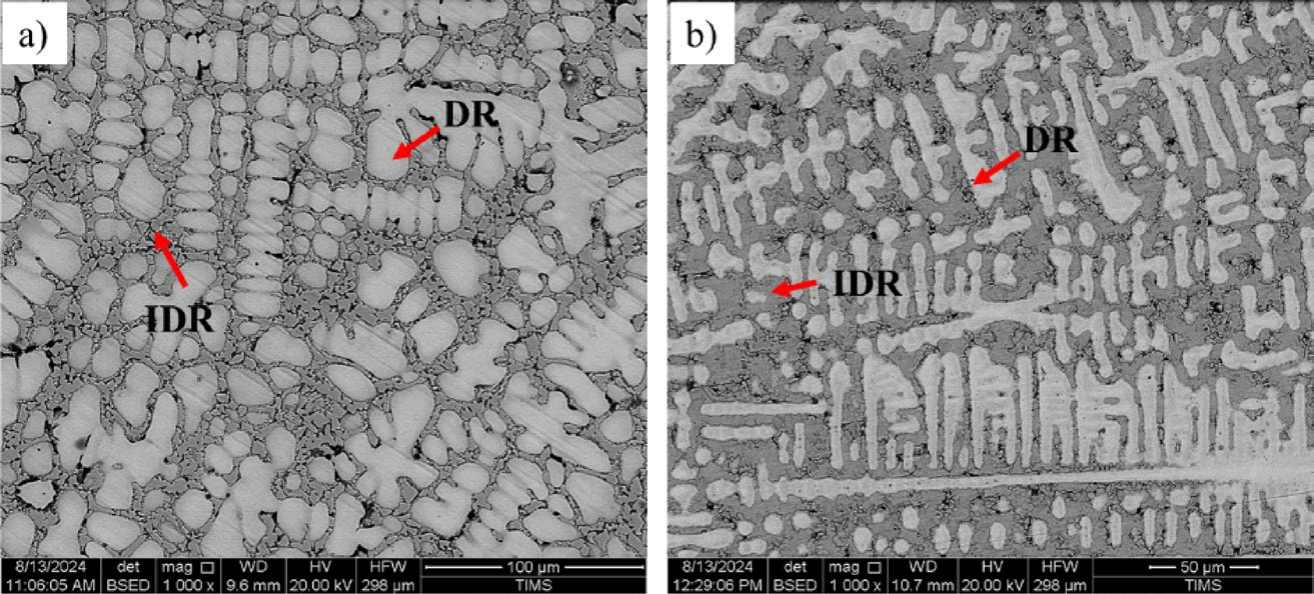
The SEM images of (**a**) 20Nb and (**b**) 10Fe10Nb CCAs.

**Fig. 8:**
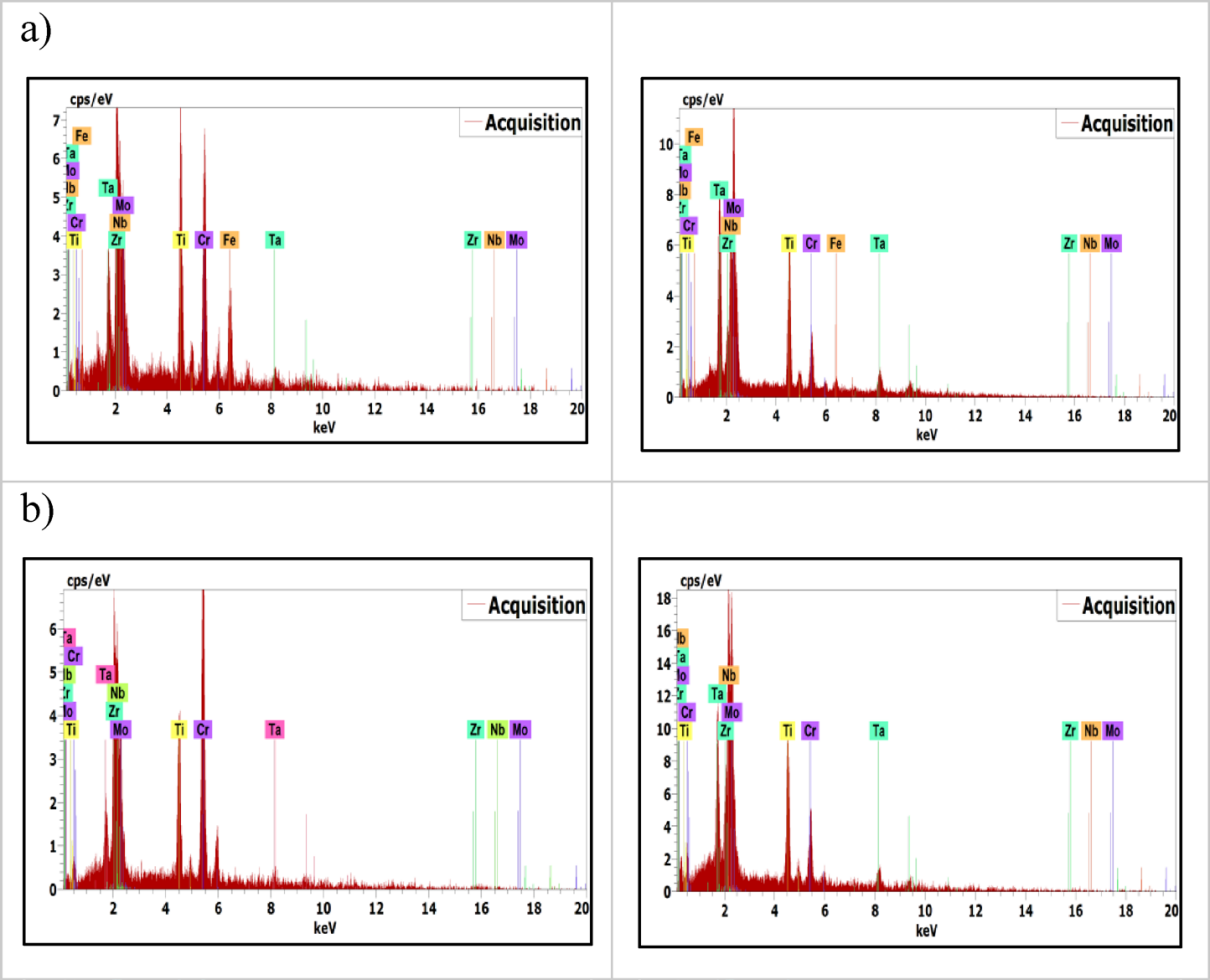
EDX point analysis of (**a**) 20Nb, and (**b**) 10Fe10Nb CCAs at spot 1 (left-IDR) and spot 2 (right-DR).

**Fig. 9:**
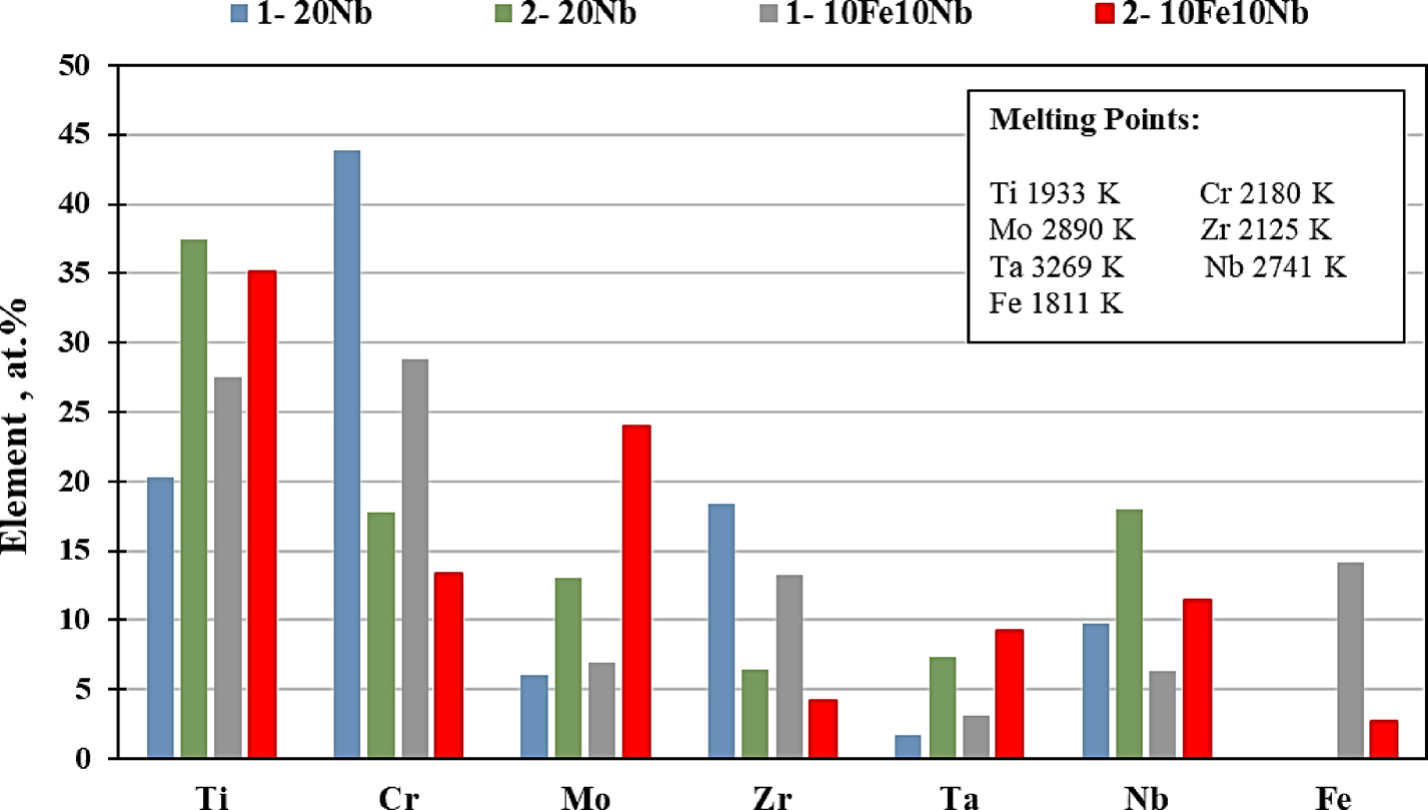
Distribution of the elements in the IDR (spot 1) and DR (spot 2) in all CCAs.

**Fig. 10:**
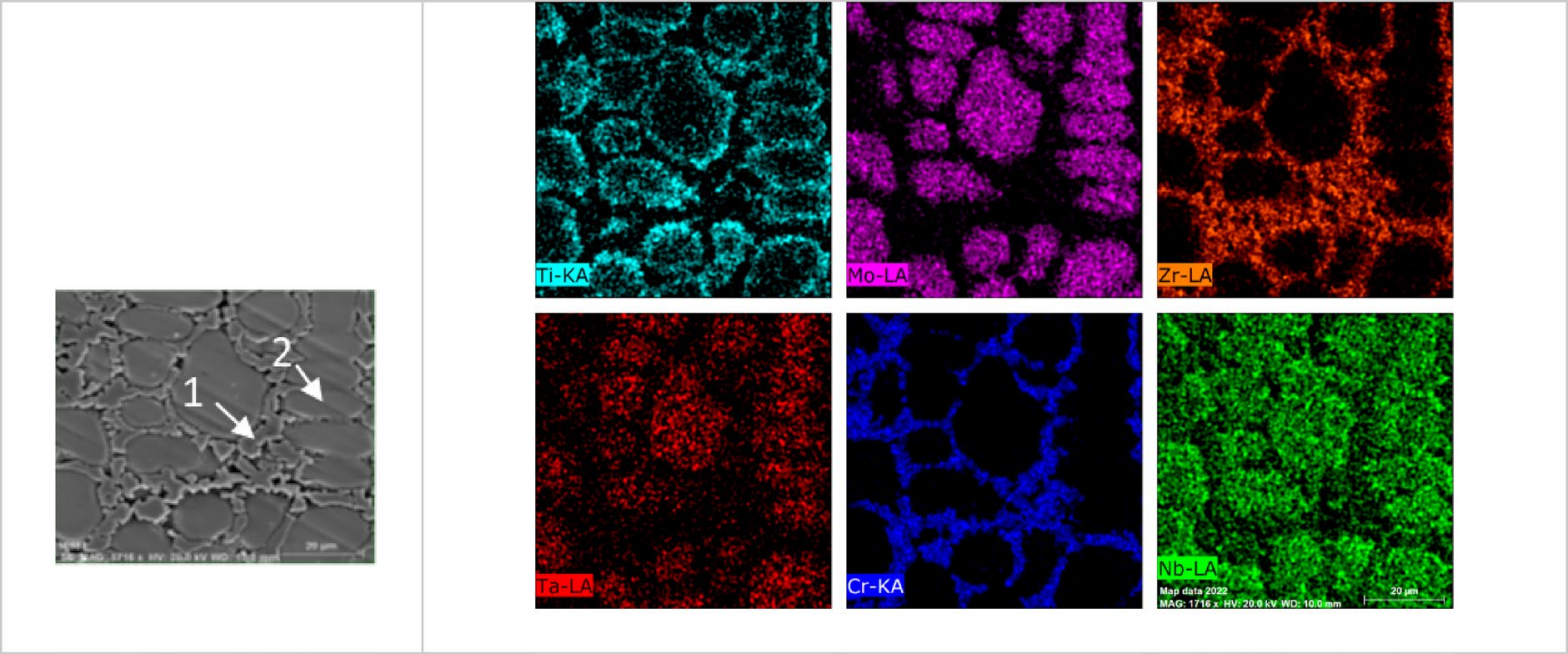
The mapping for 20Nb- CCAs with the positions of spots 1 (IDR) and 2 (DR) shown on the SEM micrograph.

**Fig. 11:**
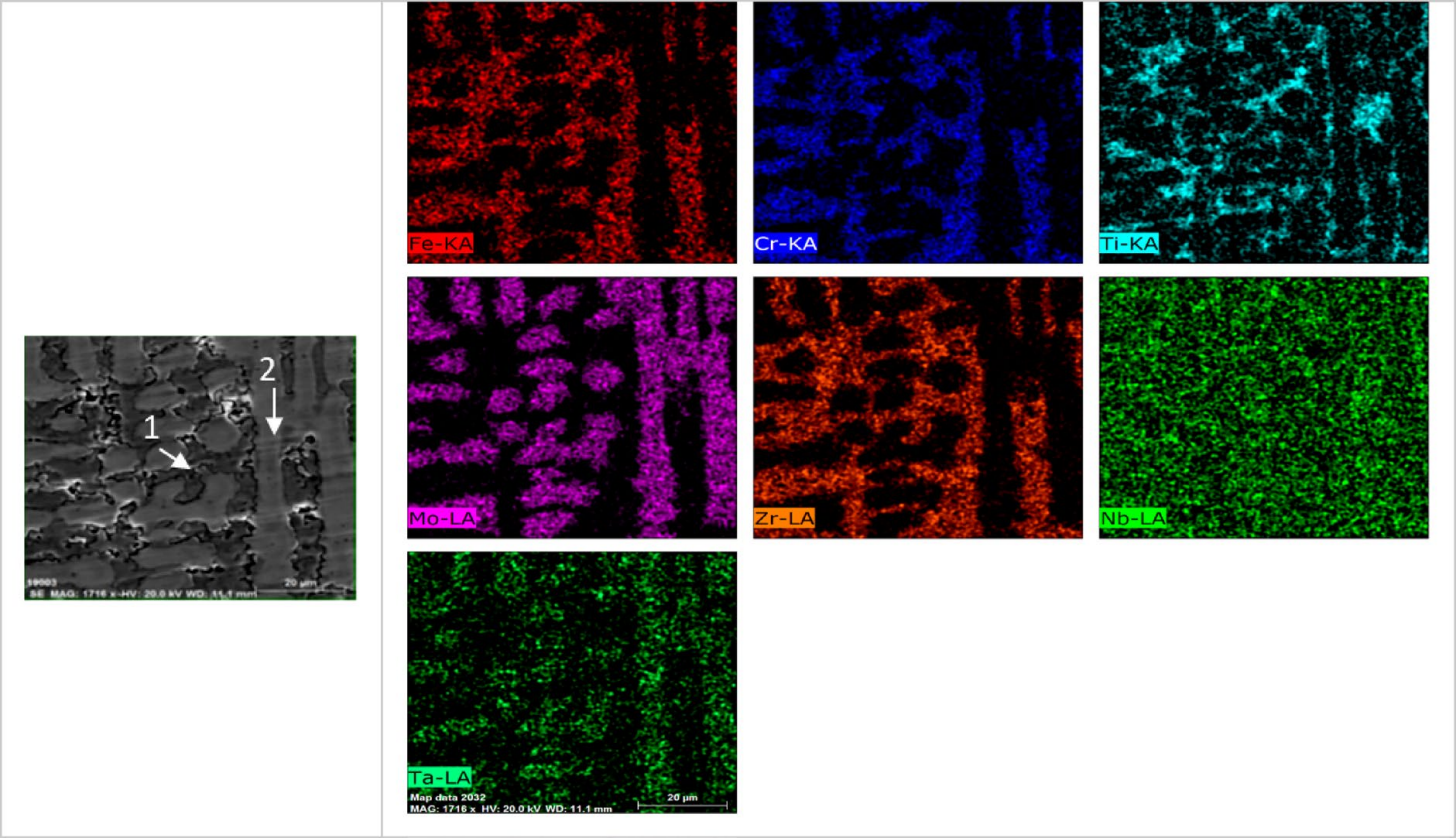
The mapping for 10Fe10Nb CCAs with the positions of spots 1 (IDR) and 2 (DR) shown on the SEM micrograph.

Figure 7 presents SEM images of the investigated CCAs, while Figs. 8 and 9 summarize the EDX point analyses of spots 1 (interdendritic region (IDR)) and 2 (dendrite arms-DR) for the microstructure of CCAs. In these micrographs, the two alloys exhibit distinct light-contrast dendrites within a continuous dark-contrast matrix. The maps of 20Nb and 10Fe10Nb CCAs are shown in Figs. 10 and 11, respectively. In these maps, both alloys exhibited microsegregation of Mo, Ta, and Nb toward the DR. The strong affinity of refractory elements for each other was evident in the formation of several intermetallic phases, such as MoNb and MoTa. Fe formed intermetallic phases with Zr & Cr, segregated to IDR, and combined with Ta & Ti, thus forming some particles in the DR, Figs. 10 and 11. It’s also remarked in the XRD patterns of Fig. 6 that the BCC-SS intensities were higher in the case of 20Nb-CCA, indicating the combined action of both Nb and Mo as efficient beta stabilizers. This stabilization efficiency was reduced in the 10Fe10Nb CCA, where more intermetallics formed, and the intensity of SS decreased significantly. The chemical formulas for 20Nb-CCA were Ti_38_Cr_18_Mo_13_Zr_6_Ta_7_Nb_18_ (DR) and Ti_20_Cr_44_Mo_6_Zr_18_Ta_2_Nb_10_ (IDR), while for 10Fe10Nb CCA were Ti_35_Cr_13_Mo_24_Zr_4_Ta_9_Fe_3_Nb_12_ (DR) and Ti_28_Cr_29_Mo_7_Zr_13_Ta_3_Fe_14_Nb_6_(IDR). These chemical formulas were estimated from the average of several EDX point analyses for each phase. This micro-segregation behavior of Mo, Ta, and Nb was explained by Wang and Xu^43^ and Juan et al.^44^, who attributed it to the preferential solidification of the high-melting-point elements into dendrite arms, thereby expelling lower-melting-point elements towards the interdendritic regions. These phases demonstrate the high chemical complexity of CCAs, in which solid-solution and intermetallic formation compete^27,28^.

### Mechanical properties

#### Hardness, fracture toughness, and wear resistance

As shown in Table 4, Vickers microhardness measurements reveal clear distinctions between the two CCAs, which directly correlate with their compositional and microstructural differences. The 10Fe10Nb alloy, with a hardness of 667.3 ± 5 HV, demonstrates a compromise in which a fine-grained mixture of Fe- and Nb-derived phases produces a synergistic strengthening effect. In contrast, the 20Nb- CCA registers coarser dendrites and hence a lower hardness of 584.2 ± 6 HV. Although 20Nb CCA has a higher percentage of refractory elements, which are supposed to offer excellent high-temperature stability^25,27^, they contributed less to room-temperature hardness due to lower intrinsic lattice strain and a scarcity of hard intermetallic phases^15^.

**Table 4:** Microhardness and fracture toughness of cast CCAs.

	20Nb	10Fe10Nb
Hardness, Hv	584.2 ± 6	667.3 ± 5
Indentation		
${K}_{IC}$, MPa. (${m}^{0.5}$)	0.52	0.85

To evaluate the fracture toughness of these alloys, the microhardness indentation method, as described by Nihara et al.^35^, was employed. This approach indirectly estimates impact or fracture toughness by analyzing crack formation around the indentation site. It is especially useful for brittle and hard materials, where standard fracture toughness tests are difficult to perform. When Palmqvist-type cracks form during indentation, their geometry provides a reliable basis for calculating fracture toughness using the empirical relationship^35^:

6 $${K}_{IC}=0.035\times {Q}^{-3/5}\times H\times (\frac{d/2}{\sqrt{l}})\times (H/E{)}^{-2/5}$$

where *K*_IC_: the fracture toughness, *Q*: a constraint factor according to^35^, *H*: the average microhardness, *E*: the Young’s modulus, *d*: the diagonal length (of indentation), and *l*: the total crack length extending from the indent corners. The validity of this approach relies on the formation of Palmqvist-type cracks, defined as short, radial cracks that propagate from the indentation edges.

The compositional dependence of mechanical performance is further demonstrated by the fracture toughness (K_IC_) data in Table 4. 20Nb-CCA exhibits a lower K_IC_ (0.515 MPa·m^1/2^), consistent with its brittle character and the high concentration of refractory intermetallics, which severely restrict plastic deformation. The 10Fe10Nb-CCA, with a K_IC_ of 0.846 MPa·m^1/2^, represents an intermediate case in which the introduction of Fe-stabilized ductile phases within the Fe–Nb microstructure facilitates partial plastic deformation at crack tips, thereby reducing stress intensity and improving toughness relative to the 20Nb alloy.

Table 5 summarizes the wear test data of the investigated CCAs. Analysis of the results reveals a clear performance ranking. Despite its relatively low hardness, the 20Nb-CCA exhibited better overall wear resistance, with the lowest specific wear rate (0.29 × 10^−10^ mm^3^/N·m), a low COF (0.57), and minimal weight loss (0.01 g). Possibly, its stable refractory-phase structure contributed to effective resistance to material removal. The 10Fe10Nb-CCA, despite its higher COF (0.68) and friction force (40.21 N), maintained a moderate wear rate of (1.07 × 10^−10^ mm^3^/Nm).

**Table 5:** Wear test data of cast 20Nb and 10Fe10Nb CCAs.

Code	Friction Force, N	weight loss, g	COF	SWR*10^10^, mm^3^/N. m
20Nb	33.74	0.01	0.57	0.29
10Fe10Nb	40.21	0.04	0.68	1.07

The integrated analysis of microhardness and fracture toughness demonstrates a fundamental strength-ductility trade-off among the two alloys. The 20Nb-CCA, while recording relatively better wear resistance, is brittle due to its intermetallic-rich structure. In contrast, the 10Fe10Nb-CCA finds an optimal middle ground, offering moderate hardness alongside high fracture toughness. This balance improves resistance to impact and to corrosion-assisted cracking, a benefit likely stemming from its fine two-phase dispersion, which enhances stress distribution and reduces galvanic corrosion. Therefore, 10Fe10Nb CCA is a promising candidate for applications requiring balanced wear and toughness properties.

#### Young’s modulus

Young’s modulus and Poisson’s ratio values shown in Table 6 reveal distinct elastic properties among the cast CCAs. Poisson’s ratios fall around 0.3 for the two alloys, confirming their good elastic recovery. The 20Nb-CCA exhibited a modulus of (85.92 GPa), which increased to 102.47 GPa in 10Fe10Nb CCA. These physically measured elastic moduli are in accordance with the previously discussed consideration of the Bo-Md diagram of Abdel-Hady et al.^42^, where a higher E modulus was expected for the 10Fe10Nb alloy in accordance with its Bo and Md values, shown in Table 2. On the other hand, the lower modulus of 20 Nb CCA, compared to the commercial Ti6Al4V alloy (~ 114 GPa)^29^, suggests it as a good candidate for bio-implantology.

**Table 6:** Young’s modulus and Poisson’s ratio of cast 20 Nb- and 10Fe10Nb CCAs.

Code	Young’s modulus, GPa	Poisson’s ratio
20Nb	85.92	0.323
10Fe10Nb	102.47	0.332

#### Electrochemical behavior

**Fig. 12:**
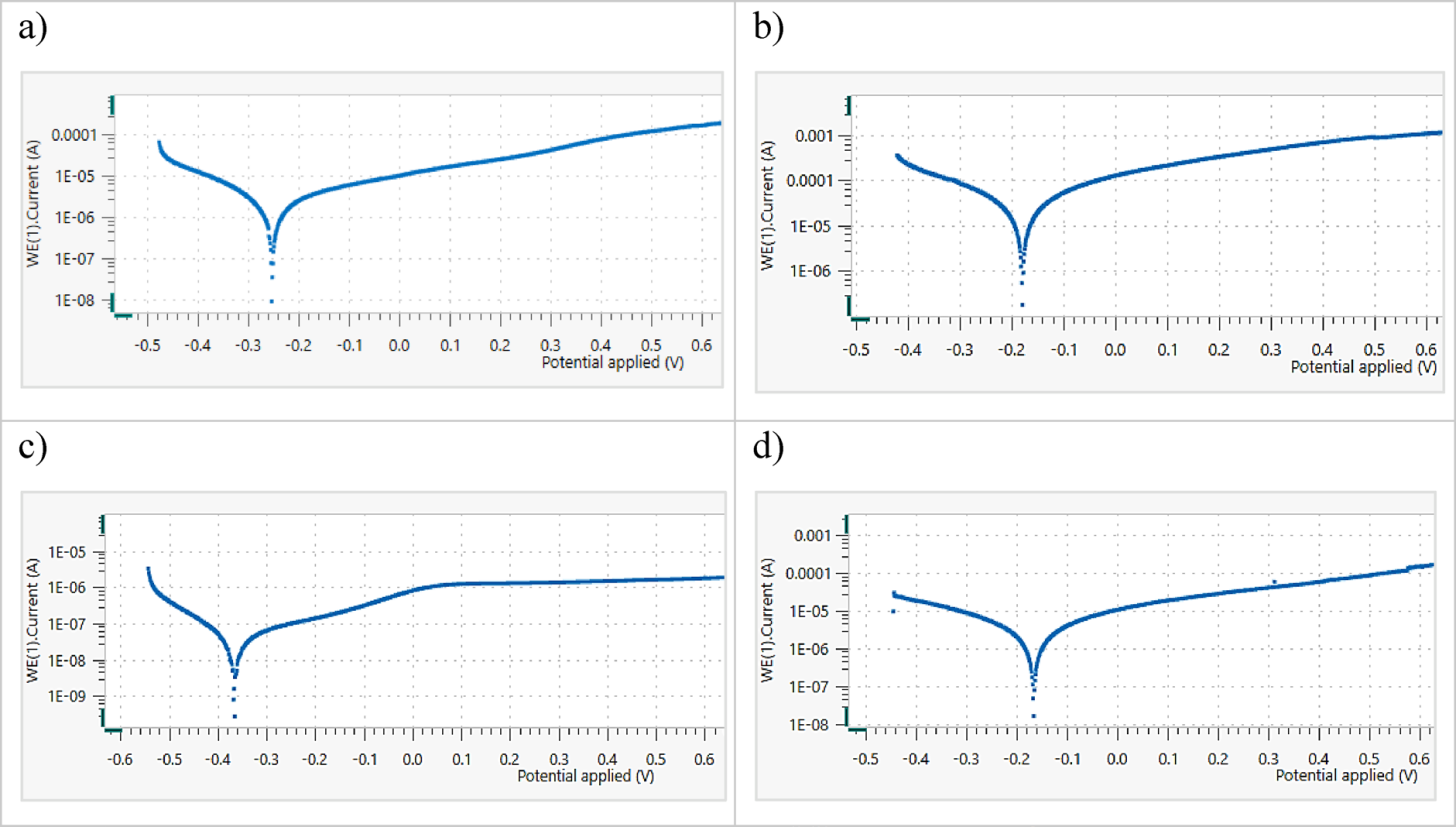
The PDP curves of 20Nb CCA in saline with (**a**) 0.0, (**b**) 1g, (**c**) 2g, and (**d**) 3g HA inhibitor at RT.

The PDP behavior of the CCAs in saline solution at room temperature was investigated, highlighting the influence of the HA inhibitor on their electrochemical corrosion behavior. The results for 20Nb-CCA, shown in Fig. 12 and Table 7, exhibit excellent corrosion resistance in saline solution, a property that was further improved by the addition of HA. The corrosion rate without HA addition was 39.09 μm/y, then declined sharply to 2.23 μm/y and 1.94 μm/y at 2 g and 3 g HA, respectively. The corrosion current also decreased drastically from 1.827 µA/cm^2^ without HA down to 0.09 µA/cm^2^ at 3 g HA, confirming a significant reduction in electrochemical activity. The incorporation of Nb promoted the formation and stabilization of a passive layer composed of Nb_2_O_5_ and NbO_2_, while the addition of HA further enhanced the film’s thickness and compactness^45^. This combined protection mechanism effectively reduced chloride-induced degradation and suppressed pitting initiation.

**Table 7:** The electrochemical parameters of 20Nb-CCAs in saline with different HA inhibitor additions at RT.

HA, g	E_corr_, V	I_corr_, µA/cm^2^	βa, V/dec	βc, V/dec	CR, µm/y
0	− 0.252	1.827	0.275	0.175	39.09
1	− 0.178	1.287	0.013	0.013	27.54
2	− 0.365	0.104	0.470	0.128	2.23
3	− 0.165	0.090	0.009	0.010	1.94

**Fig. 13:**
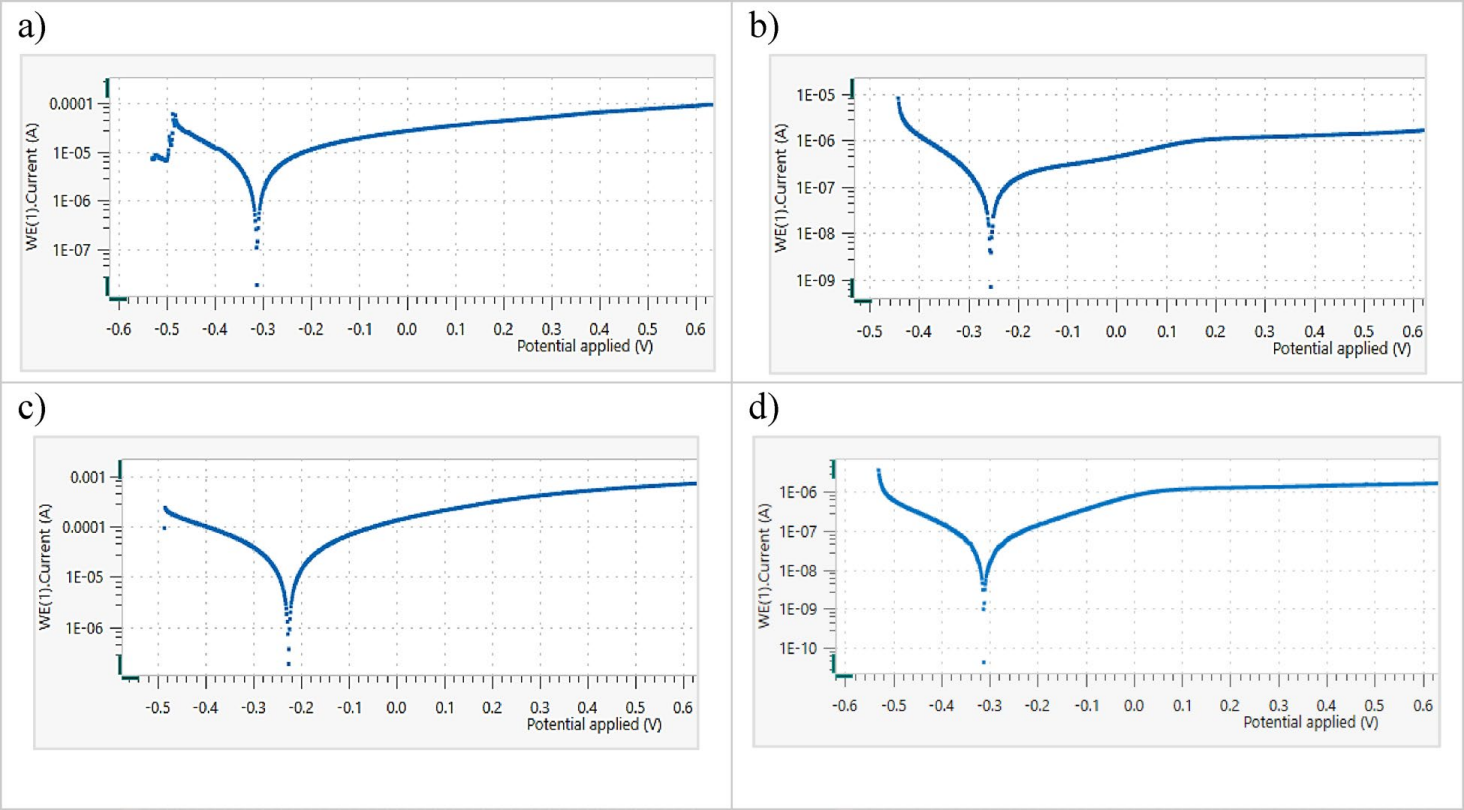
The PDP curves of 10Fe10Nb CCA in saline with (**a**) 0.0, (**b**) 1g, (**c**) 2g, and (**d**) 3g HA inhibitor at RT.

In 10Fe10Nb-CCA samples, as shown in Fig. 13 and Table 8, the corrosion rate in saline without HA was relatively high (61.84 μm/y), whereas the addition of HA significantly mitigated corrosion. At 1 g HA, the corrosion rate dropped to 13.75 μm/y, and at 2 g HA, it then decreased further to 11.14 μm/y. The best protection was achieved at 3 g HA, where CR = 0.38 μm/y and Icorr = 0.02 µA/cm^2^, indicating near-passivation. The relative negative shift in Ecorr and reduction in Icorr with increasing HA concentration demonstrate the barrier effect of HA particles. These results confirm that 10Fe10Nb-CCAs can achieve strong corrosion inhibition in saline when adequately passivated with HA. Both CCAs exhibited protective oxide films containing TiO and Ti_2_O₃, thereby improving corrosion resistance^46^.

**Table 8:** The electrochemical parameters of 10Fe10Nb-CCAs in saline with different HA inhibitor additions at RT.

HA, g	E_corr_, V	I_corr_, µA/cm^2^	βa, V/dec	βc, V/dec	CR, µm/y
0	− 0.311	2.72	0.163	0.127	61.84
1	− 0.255	0.60	2.252	0.126	13.75
2	− 0.225	0.49	0.005	0.007	11.14
3	− 0.311	0.02	0.091	0.067	0.38

### Surface morphology

**Fig. 14:**
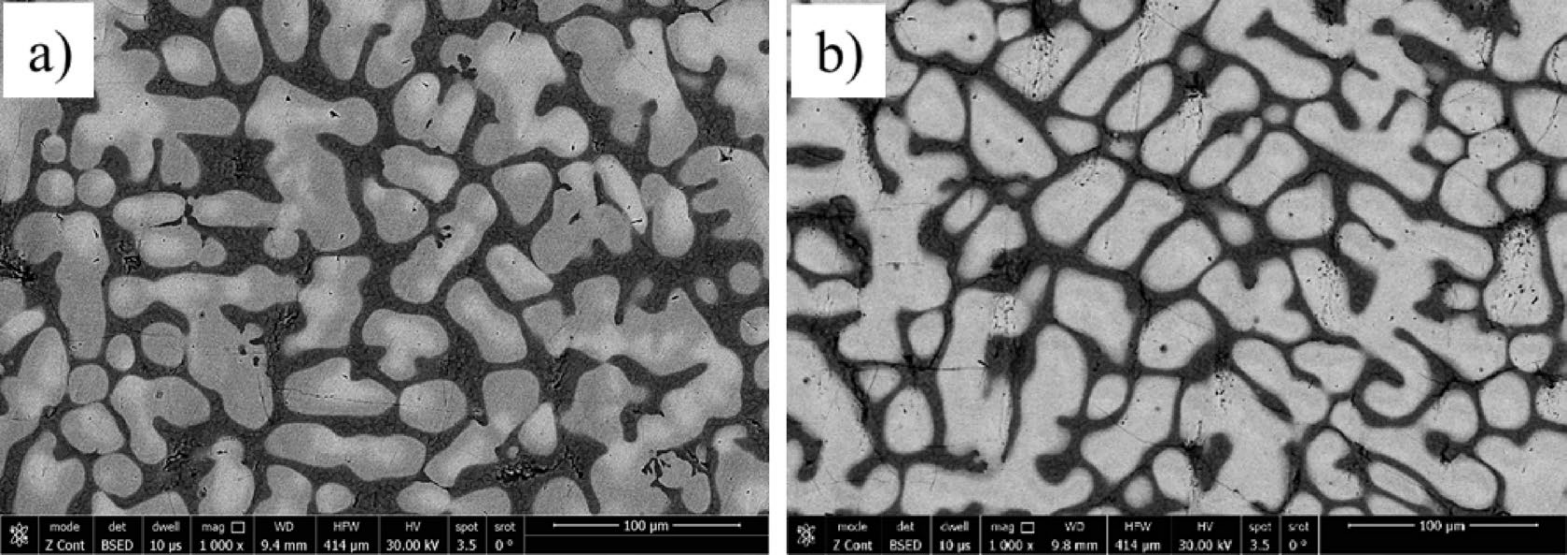
The SEM images of the corroded 20Nb-CCA at (**a**) 0.0 HA and (**b**) 3 g HA.

**Fig. 15:**
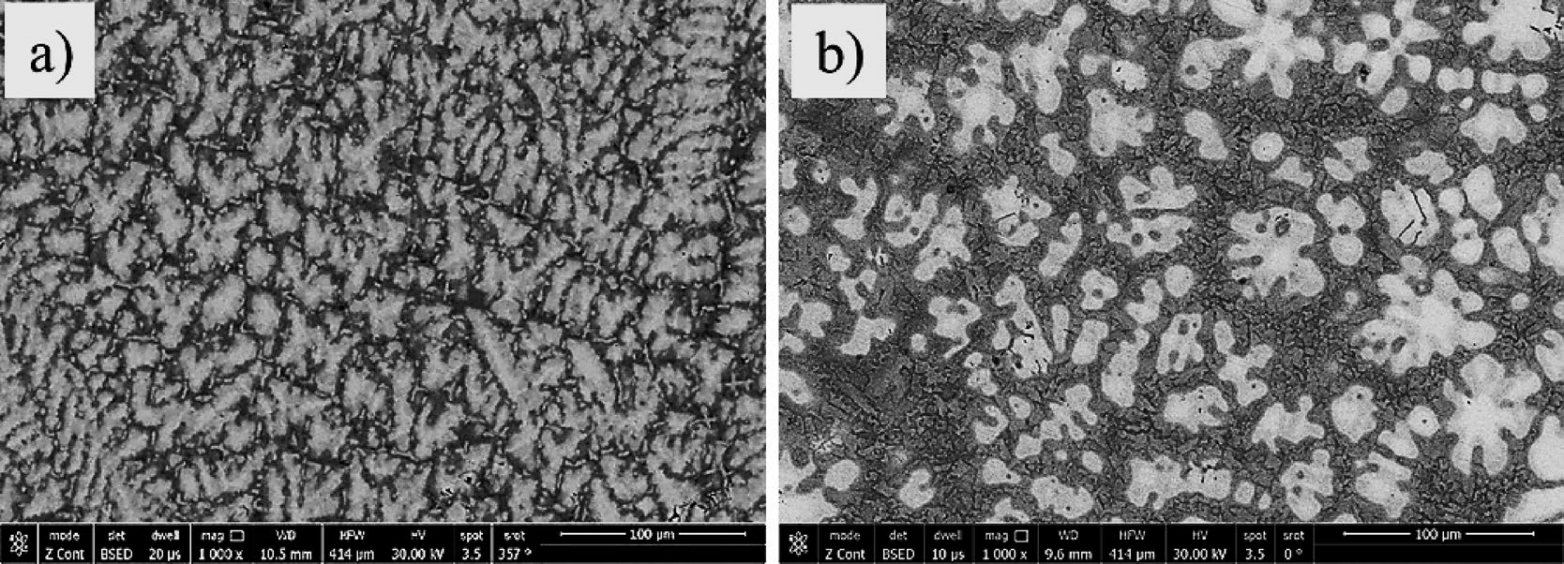
The SEM images of the corroded 10Fe10Nb-CCA at (**a**) 0.0 HA and (**b**) 3 g HA.

The SEM micrographs in Fig. 14 illustrate the surface morphologies of the corroded 20Nb-CCAs samples after immersion in saline solution. In the absence of HA (0.0 g), the 20Nb-CCAs surface exhibited localized corrosion features, with relatively rough, irregular areas formed after exposure to the saline environment, Fig. 14a. The surface appears to contain corrosion products distributed non-uniformly across the CCAs, leading to galvanic corrosion, as well as uniform and localized (pitting) corrosion. However, when 3 g of HA was added (Fig. 14b), the sample showed a smoother, more compact surface morphology. This indicates the formation of a protective layer that partially covers the CCA surface. The presence of HA appears to reduce the severity of corrosion attack and contribute to surface stabilization. A similar trend was observed for the 10Fe10Nb-CCA, although the corrosion features are slightly more pronounced due to the presence of Fe. Without HA, the 10Fe10Nb-CCA exhibited pits and corrosion products typical of chloride-containing environments (Fig. 15a). The addition of HA resulted in a more uniform corrosion product layer (Fig. 15b). These observations suggest that HA plays a beneficial role in modifying the corrosion behavior of the CCAs investigated.

**Fig. 16:**
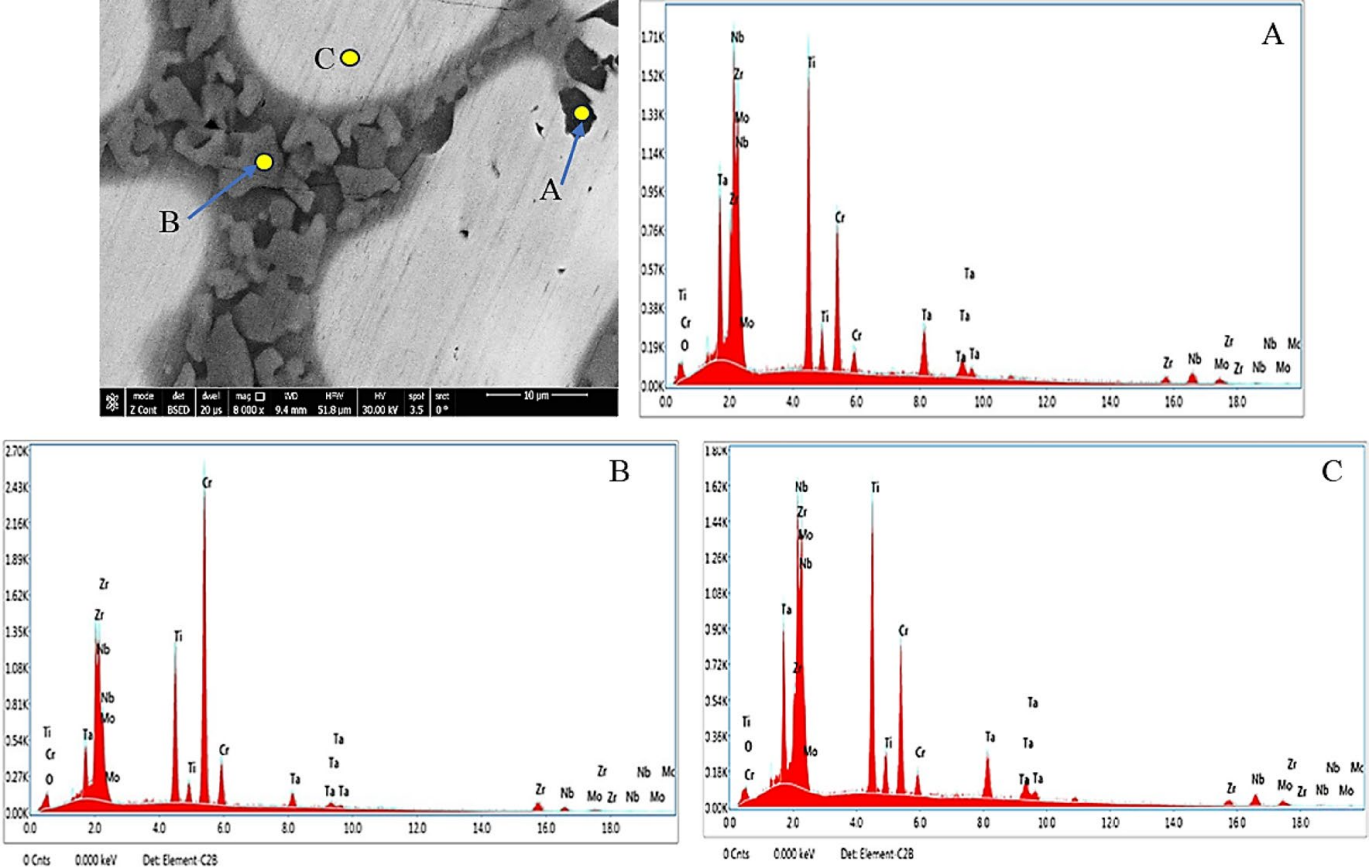
A SEM micrograph and the corresponding EDX analysis of points (**A**, **B**, and **C**) of the corroded 20Nb-CCA in saline at 0.0 HA.

**Fig. 17:**
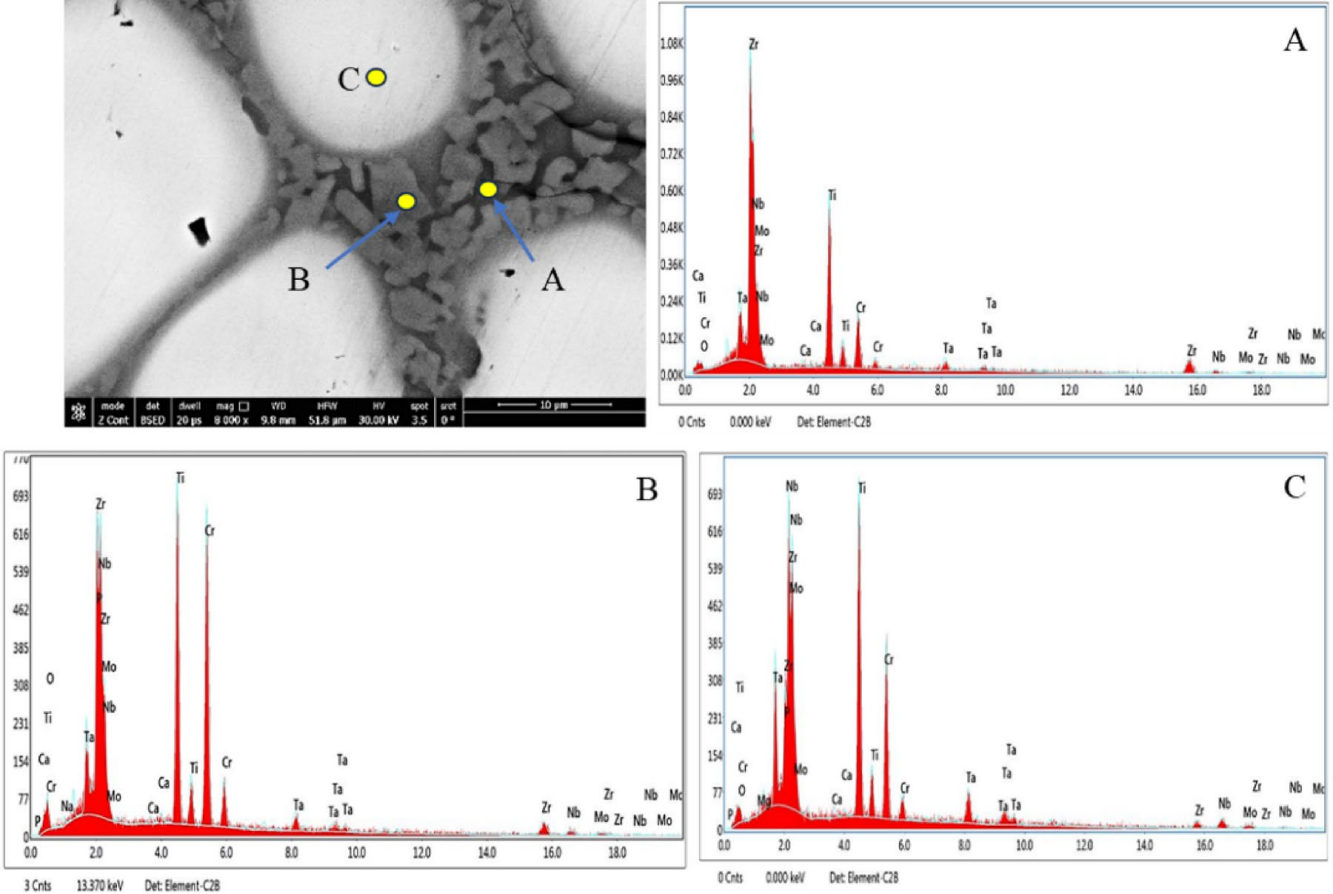
A SEM micrograph and the corresponding EDX analysis of points (**A**, **B**, and **C**) of the corroded 20Nb-CCA in saline at 3 g HA.

Figures 16 and 17 show the EDX analyses of the corroded 20Nb-CCAs in saline, without and with HA, respectively. The EDX results for spots A-C (Table 9) reveal that the CCA surface mainly consists of the base elements Ti, Cr, Mo, Zr, Ta, and Nb, along with a noticeable amount of oxygen. The presence of oxygen indicates the formation of oxide films during corrosion exposure. In the absence of HA (Fig. 16), elements such as Ca, Mg, Na, and P were not detected, confirming that the corrosion products are mainly metallic oxides originating from the CCA itself. The relatively high Nb and Ti contents in the analyzed spots suggest that these elements contribute to the stability of the passive film. When HA is introduced into the saline solution (Fig. 17), additional elements, including Ca and small amounts of P and Mg, appear in the EDX spectra. These elements originated from the HA particles present in the solution. The detection of Ca and P confirms the adsorption or deposition of HA-related compounds on the CCA surface. This deposited layer may enhance the protective properties of the passive film. Consequently, the presence of HA appears to promote the formation of a composite oxide/HA layer, thereby improving corrosion resistance.

**Table 9:** The EDX results of corroded 20Nb- and 10Fe10Nb-CCAs in at.%.

Code	Spot No	Elements at. %												
		O	Cl	Ca	Mg	Na	P	Ti	Cr	Mo	Zr	Ta	Nb	Fe
20Nb 0.0 HA	A	13.8	0.0	0.0	0.0	0.0	0.0	27.3	15.1	12.9	7.7	4.5	18.7	–
	B	9.9	0.0	0.0	0.0	0.0	0.0	16.7	39.4	6.2	14.9	1.0	10.6	–
	C	9.9	0.0	0.0	0.0	0.0	0.0	27.3	16.2	13.9	7.4	4.4	20.8	–
20Nb 3 g HA	A	11.3	0.0	1.2	0.0	0.0	0.0	26.8	10.0	4.9	32.0	2.0	11.6	–
	B	20.4	0.0	0.5	0.0	2.8	0.0	23.2	24.9	4.9	13.3	1.5	8.5	–
	C	8.3	0.0	1.0	1.7	0.0	0.4	29.8	17.8	12.2	8.7	3.2	16.9	–
10Fe10Nb 0.0 HA	A	7.14	0.08	0.0	0.0	1.1	0.0	16	16.4	14.8	14.2	11.8	5.03	13.5
	B	22.9	0.1	0.0	0.0	2.4	0.0	22.2	15.7	8.5	7.2	2.9	3.9	14.2
	C	8.4	0.3	0.0	0.0	3.4	0.0	18.6	23.7	9.5	10.5	5.2	4.4	15.9
10Fe10Nb 3 g HA	A	24.8	0.0	0.3	0.0	0.0	0.9	30.5	14.6	3.2	17.0	0.8	7.9	0.2
	B	16.1	0.0	0.3	0.0	0.0	0.1	14.0	39.3	5.5	14.2	1.2	8.8	0.5
	C	20.7	0.0	0.7	0.0	0.0	0.1	20.6	9.9	16.1	5.1	5.8	20.5	0.5

**Fig. 18:**
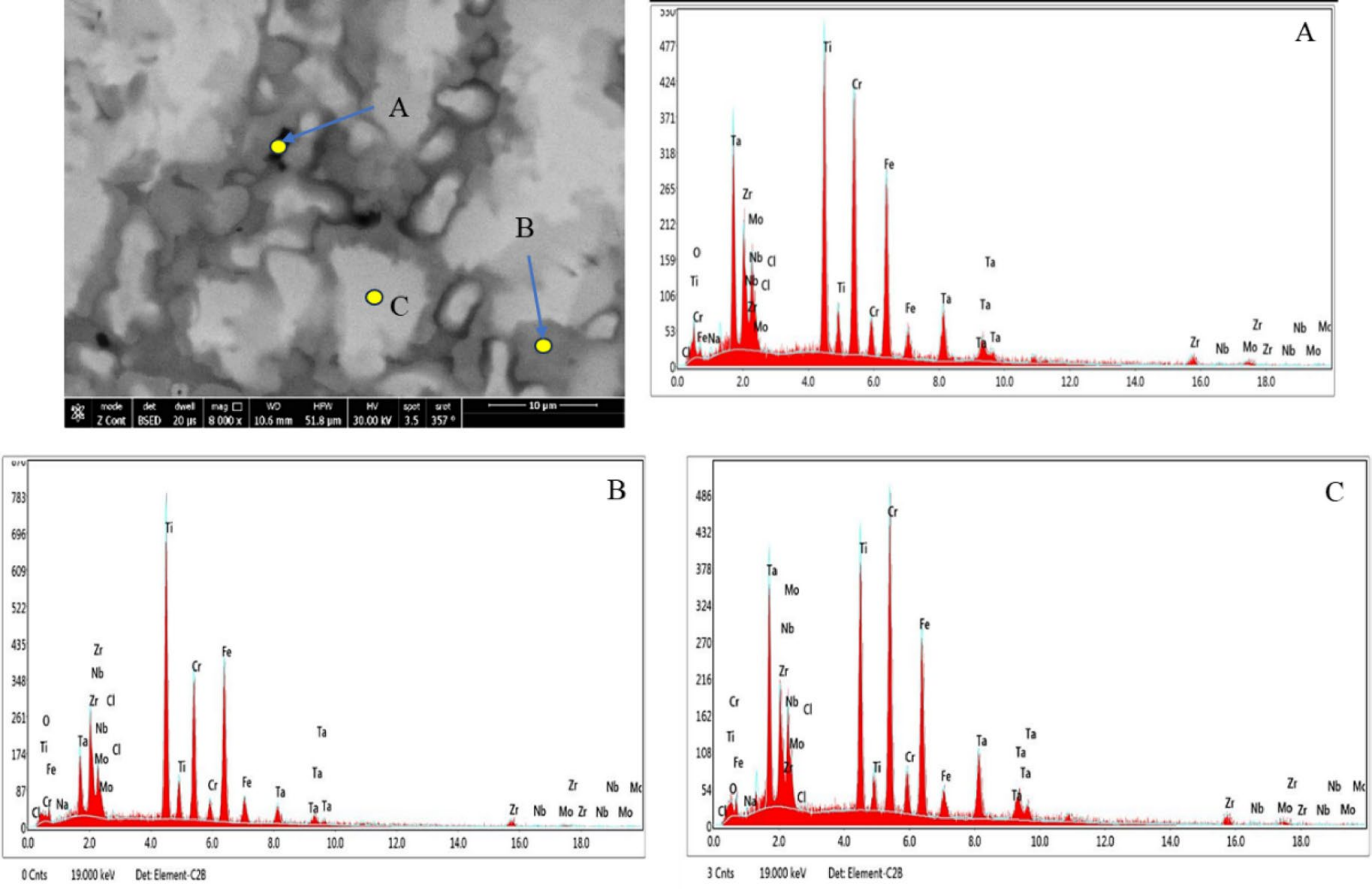
A SEM micrograph and the corresponding EDX analysis of points (**A**, **B**, and **C**) of the corroded 10Fe10Nb-CCA in saline at 0.0 g HA.

**Fig. 19:**
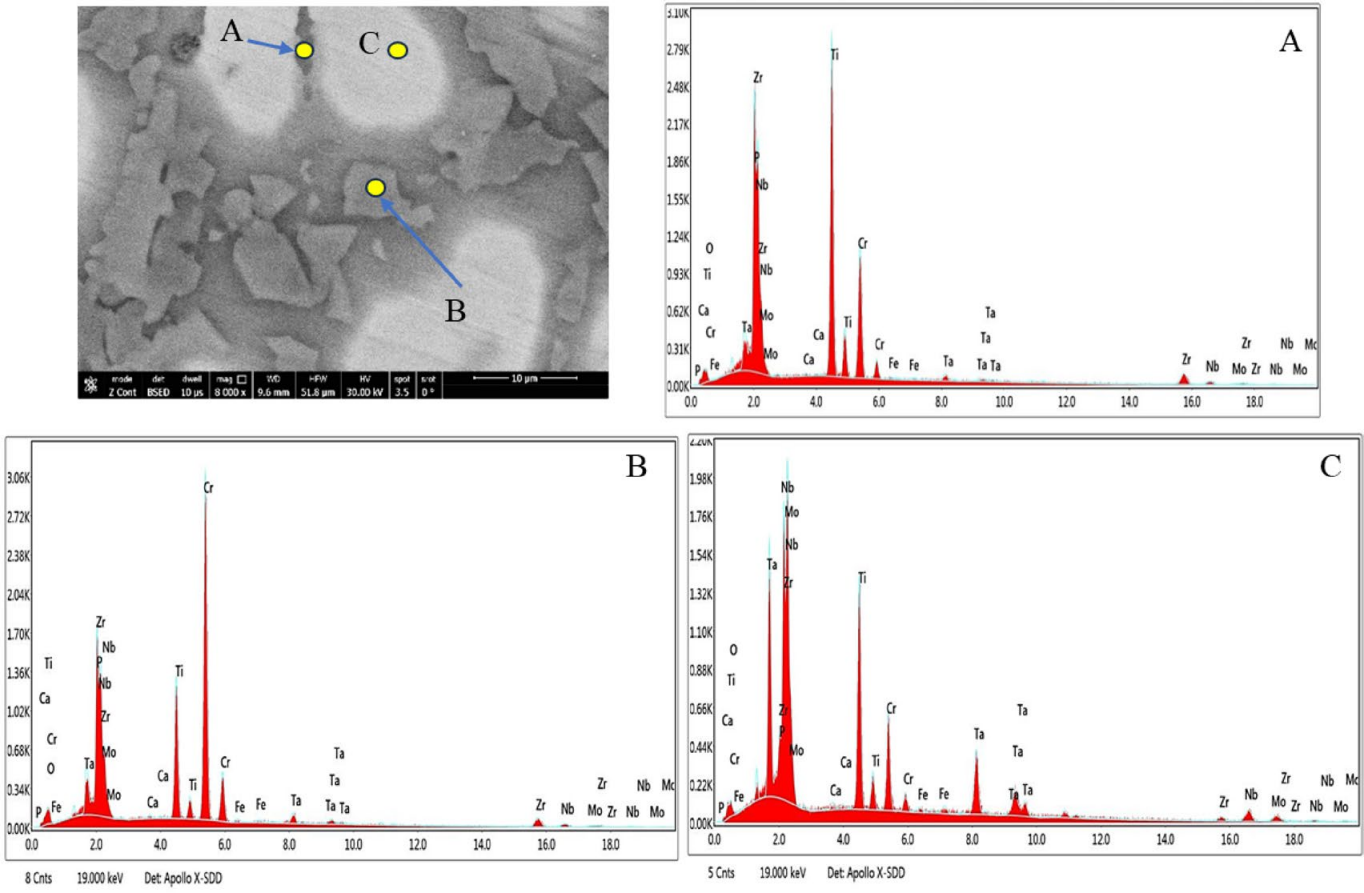
A SEM micrograph and the corresponding EDX analysis of points (**A**, **B**, and **C**) of the corroded 10Fe10Nb-CCA in saline at 3 g HA.

Figures 18 and 19 present the EDX analyses of the 10Fe10Nb-CCAs in saline, both with and without HA. The EDX data for A-C spots in Fig. 18 show that the surface contains oxygen along with the main alloying elements, including Ti, Cr, Mo, Zr, Ta, Nb, and Fe. The relatively high oxygen content suggests the formation of oxide corrosion products during immersion in the saline solution. Small amounts of Na and Cl are also detected, indicating the interaction between the CCA surface and the chloride-containing electrolyte. The presence of Fe in this CCA may influence corrosion behavior and contribute to the formation of different oxide phases. When 3 g HA is added to the saline solution (Fig. 19), the EDX spectra show traces of Ca and P, which are characteristic of HA. The oxygen content also increases, suggesting the formation of a thicker or more complex oxide layer. Furthermore, the Fe content decreases significantly in the analyzed spots, which may indicate surface coverage by corrosion products and HA deposits. The combination of oxides and HA led to the formation of a protective surface layer. This layer likely improves corrosion resistance and enhances the bioactivity of the CCA surface in saline.

The PDP results, along with the surface characterization, indicate that the addition of HA significantly enhances corrosion resistance. This improvement is supported by SEM observations, which reveal a smoother, more compact surface morphology in the presence of HA. Furthermore, EDX analysis detected Ca and P elements on the CCA surface, confirming the deposition of HA-related compounds and the formation of a protective HA-containing layer. This behavior suggests that HA adsorbs onto the CCA’s surface and forms a barrier layer that limits access of corrosive species and suppresses the electrochemical reactions responsible for metal dissolution. The electrochemical results are consistent with the surface analyses, as SEM micrographs show fewer corrosion defects and a more uniform surface film. At the same time, EDX spectra confirm the presence of Ca and P derived from HA. These findings indicate the formation of a protective composite layer composed of metallic oxides and HA-derived compounds, thereby reducing interactions between the CCA surface and the chloride-containing saline environment. Thus, the combined electrochemical and microstructural results demonstrate that increasing HA concentration significantly improves the corrosion resistance of both 20Nb-CCAs and 10Fe10Nb-CCAs, with the highest protection observed at 3 g HA.

## Conclusions

This study comprehensively evaluated the relationship between composition, microstructure, and performance of two arc-melted Ti-based CCAs: Ti_30_Cr_20_Mo_15_Zr_10_Ta_5_Nb_20_, and Ti_30_Cr_20_Mo_15_Zr_10_Ta_5_Nb_10_Fe_10_. The following conclusions were drawn:

- The prepared CCAs formed BCC1 as the main SS and minor BCC2, along with some intermetallic phases. Partial replacement of Nb by Fe decreased the SS intensity and allowed more intermetallic compounds. In both CCAs, the microstructure was dendritic, with the high-melting-point elements segregated in the dendritic regions.
- 20Nb CCA showed a relatively lower hardness and better wear resistance due to its stable refractory-phase structure that possibly resists the material removal.
- 10Fe10Nb CCAs showed a higher Young’s modulus of (102.47 GPa) compared to (85.92 GPa) for 20Nb-CCA.
- Both CCAs showed excellent corrosion resistance in the presence of 3 g of HA inhibitor, where the corrosion rate of 20Nb decreased from 39.09 μm/y at 0.0 HA to 1.94 μm/y at 3 g HA, and that of 10Fe10Nb reduced from 61.84 μm/y at 0.0 g HA to 0.38 μm/y at 3 g HA. This demonstrates the effective barrier provided by HA particles. Moreover, the incorporation of Nb promoted the formation and stabilization of a passive layer composed of Nb_2_O_5_ and NbO_2_, while the addition of HA further enhanced the film’s thickness and compactness.

In summary, the properties of CCAs can be tailored, and lower-cost versions can be produced through elemental control to achieve a microstructure that relies on solid solutions while retaining some intermetallic phases, meeting specific property requirements for certain applications. Future work investigating cytotoxicity, ion release, and long-term immersion in simulated body fluid is recommended to validate the potential of the developed CCAs for biomedical use.

## Data Availability

All data generated or analyzed during this study are included in this published article‎.
